# Dysbiosis of the gut microbiome is a risk factor for osteoarthritis in older female adults: a case control study

**DOI:** 10.1186/s12859-021-04199-0

**Published:** 2021-06-03

**Authors:** Juanjuan Chen, Anqi Wang, Qi Wang

**Affiliations:** 1 Cuiying Biomedical Research Center, Lanzhou University Second Hospital, Lanzhou, 730030 Gansu People’s Republic of China; 2 Institute of Clinical Research and Translational Medicine, Gansu Provincial Hospital, Lanzhou, Gansu People’s Republic of China

**Keywords:** Older females, Osteoarthritis, Gut Microbiome, Compositional and functional alterations, Classifier model

## Abstract

**Background:**

Osteoarthritis (OA) is a multifactorial joint degenerative disease with low-grade inflammation. The gut microbiome has recently emerged as an pathogenic factor of OA, and prebiotics supplementation could alleviate OA symptoms in animal models. However, the relationship between the gut microbiome and OA in the older female adults is hitherto not clear.

**Results:**

Here we studied the gut microbiome of 57 OA patients and their healthy controls by metagenome-wide association study based on previously published data. A significant reduction in the richness and diversity of gut microbiome were observed in OA patients. *Bifidobacterium longum* and *Faecalibacterium prausnitzii* were decreased while *Clostridium* spp. was increased in the OA group. The functional modules, particularly the energetic metabolism and acetate production were also decreased in the OA patients. To evaluate the diagnostic value of identified species for elderly patients with OA, we constructed a set of random forest disease classifiers based on species differences between the two groups. Among them, 9 species reached the lowest classification error in the random forest cross validation, and the area under ROC of the model was 0.81.

**Conclusions:**

Significant alterations in the gut microbial composition and function were observed between the older patients with OA and their controls, and a random forest classifier model for OA were constructed based on the differences in our study. Our study have identified several potential gut microbial targets in the elderly females with OA, which will facilitate the treatment of OA based on gut microbiota, is of great value in alleviating pain and improving the quality of life for them.

**Supplementary Information:**

The online version contains supplementary material available at 10.1186/s12859-021-04199-0.

## Background

Osteoarthritis (OA) is a degenerative joint disease resulting in joint degeneration, inflammation, pain, stiffness and disability [1], clinically manifested by joint pain and stiffness [2]. OA is recognized as a disease with complex causes including genetic and epigenetic factors, sex, age, obesity, and sedentary lifestyle [3], and affecting about 3.3% people of the world population [4]. Among individuals aged over 60 years old, almost 10% of males and 18% of females are affected [5]. Although efforts are being made to develop effective treatments, the only accepted and available clinical approaches was palliation. Recent studies have demonstrated that an imbalance of the gut microbiome (dybiosis) is an emerging pathogenic factor for OA in both humans and murine [6].

Gut microbial components and their metabolites are closely associated with various aspects of the host physiology including metabolism [7], immunity and inflammation [8]. Gut microbial dysbiosis has been involved in the pathogenesis of various inflammatory diseases, such as obesity [9], ankylosing spondylitis [10] and rheumatoid arthritis [11]. Early publishes suggested a correlation between the proinflammatory factors such as lipopolysaccharide (LPS) and OA in both rat models with OA [12] and OA patients [13]. Later Ulici et al. [14] found a decline in the severity of posttraumatic osteoarthritis in germ-free mice which confirmed the role of the gut microbiome in OA. Boer CG et al. [15] further corroborated the role of the gut microbiome in OA in 1427 participants enrolled in the Rotterdam study-III with hip and/or knee OA by correlating the increased WOMAC score with the abundance of proinflammtory *Streptococcus* spp. To further study the metabolic disorders in non-obese mice and the role of the gut microbiome, Guss et al. [16] applied the Toll-like receptor-5 deficient mice and found that alone it was not sufficient to induce OA, however they suggested that increased levels of LPS in HFD-fed mice was associated with higher OARSI scores and a dysbiosis of Firmicutes increase, which further clarified the gut microbial composition in OA occurrence and development. To investigate whether gut microbiome intervene can remit symptoms of OA, Schott et al. [17] found that the anti-inflammatory *Bifidobacterium pseudolongum* was reduced in obese mice with OA and oligofructose supplementation can restore the dysbiosis to relieve the OA in obese mice. Consistent with Schott et al. findings, Rios et al. [18] showed that a maximum protection could be achieved by combining the oligofructose supplementation with exercise in HFD-fed rats with OA, with an increase in *Bifidobacterium* and *Roseburia* and a decrease in *Clostridium leptum* and *Akkermansia muciniphila* levels. Another recent study in a guinea pig model of OA showed that oral administration of *Bifidobacterium longum* CBi0703 could reduce cartilage structural lesions and provide an overall joint protective effect [19].

The findings provided new insights into the understanding and treatment of OA based on the gut microbiome. However, the changes of the gut microbiome in the older female adults with OA were hitherto not clearly studied. It is not known whether these findings were appropriate for older female adults with OA. Therefore, the metagenome-wide association study (MWAS) was applied in this study for 57 older female OA patients’ stool samples and their BMI, age, and sex matched healthy controls’ to investigate the gut microbial alterations and their correlation with OA in the older female adults. The results showed significant changes in both microbial composition and functional modules, and a classifier of nine bacteria with area under curve (AUC) of 0.81 was constructed in our study to identify OA in the older females. These observations suggested that gut microbiota could be practical targets and supplementation of beneficial gut microbes such as *Faecalibacterium prausnitzii* (*F. prausnitzii*) and *B. longum* could be novel therapeutic strategy to treat OA in the older adults.

## Methods

### The aim, design and setting of the study

This study was performed to study the gut microbial component and functional alterations in older female adults with OA. The shotgun metagenomic sequencing data of human fecal samples were derived from the UK Twins Project [20]. All of the samples and clinical indexes were collected by Prof. Spector’s group at the Kings College London University. Subjects were excluded if they had a history of chronic serious infection, any current infection and any type of malignant cancer; individuals who had received antibiotic treatment within 1 month before participating in this study were also excluded.

### The characteristics of participants

Totally 57 patients with OA were included in this study and among them 20 were twins. Two pairs of twins were monozygotic and eight pairs were dizygotic. The mean age of these patients were 65.0 ± 7.7 years and their mean BMI was 25.9 ± 4.5 kg/m^2^. The 57 healthy controls were selected by matching the age and BMI, among them including one pair of monozygotic (MZ) twins and one pair of dizygotic (DZ) twins (Additional file 1: Table S1a).

### Statement

The cohort of this study was from the UK Twins Project [20] and we used the clean data (microbial genomes after removing the low quality and human genomes) for this study which was approved by Prof. Spector’s group at the Kings College London University.

Meanwhile, all our methods were performed in accordance with the relevant guidelines and regulations.

### Shotgun metagenomic sequencing

Metagenomic shotgun sequencing was performed on Illumina platform for human fecal samples (paired end library of 350-bp and 150-bp read length). The raw reads that had 50% low-quality bases (quality ≤ 20) or more than five ambiguous bases were excluded. The remaining reads were mapped to the human genome (hg19) by SOAP v2.22 (-m 100 - × 600 -v 7 -p 6 -l 30 -r 1 -M 4 -c 0.95), and the matching reads were removed as Fang et al. reported [21]. The high-quality nonhuman reads were defined as clean reads.

### Taxonomic and KO profiling

The clear reads were aligned to the updated 11.4 million genes catalog reported by Xie et al. [20] to get the gene abundance. The relative abundance profile of KOs was determined by summing the relative abundance of genes from each KO using the mapped reads per sample. The abundance of each gut metabolic module (GMM) [22] (-a 2 -d GMM.v1.07.txt -s average) was calculated as shown in the former published articles. To obtain the taxonomic profiles, Metaphlan2 [23] (- input_type fastq - ignore_viruses - nproc 6) was used to generate phyla, genera, and species profiles from the clean reads.

### Functional modules prediction

The gut metabolic modules (GMMs) reported by Vieira-Silva et al. [24] were used in our study to study microbial functional changes. Differentially enriched KEGG modules were identified according to their reporter score from the Zscores of individual KOs. Each differential GMM’s abundance was calculated as the median of KO abundance with 66% coverage just as showed in the former article [24].

### Permutational multivariate analysis of variance

Permutational Multivariate Analysis of Variance [PERMANOVA; code: R 4.0.3:adonis (dist ~ phe, permutations = 10,000)] was performed based on the gut microbial gene abundance profile to study the effect of clinical indexes on the gut microbiome. BMI and OA were significantly different between two groups (Additional file 1: Table S1b).

### Diversity

Alpha-diversity [within-sample diversity, R 4.0.3:diversity(data, index = 'shannon')] was calculated using the Shannon index depending on the gene and species profile (Additional file 1).

### Metagenome-wide association study

For the identified species, genera, phyla and functional modules, the relative abundance of each was compared between the patients and controls via Wilcoxon rank-sum test followed by a Storey’s FDR correction. Moreover, taxonomic and functional modules were correlated with diagnosis via Semi-partial Spearman correlation tests (R package ppcor, code: R 4.0.3, ppcor.test [phe1, phe2, c(age,BMI), method = "spearman")] adjusting for BMI and age. The co-occurrence network for different taxons and functional modules was visualized using Cytoscape 3.4.0. Pair-wise comparison of species and functional modules between the patients and controls was conducted via paired Wilcoxon rank-sum test with a mutilple testing correction of Benjamini–Hochberg correction.

For the random forest model, five-fold cross-validation was performed ten times using the species and functional modules abundance profiles of the two groups. The test error curves from ten trials of five-fold cross-validation were averaged. We chose the model which minimized the sum of the test error and its standard deviation in the averaged curve according to the reference article [25].

## Results

### The gut microbiota profile of osteoarthritis

**Fig. 1:**
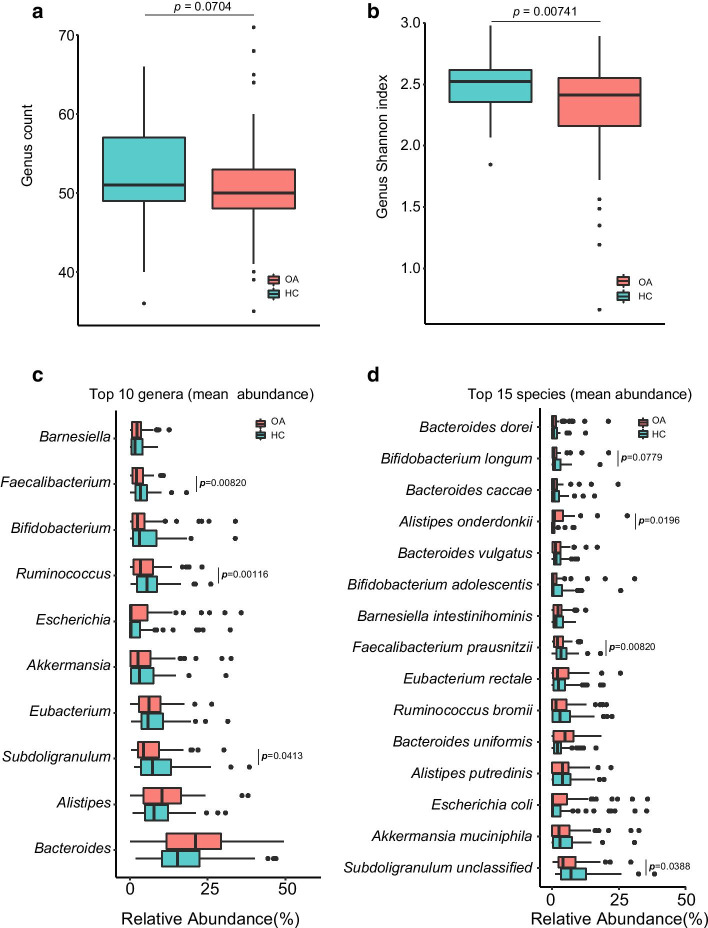
Reduced gut microbial in the older females with osteoarthritis (OA). The differences of the count (**a**) and alpha-diversity (**b**, Shannon index) at the genus levels of the OA patients and their healthy controls (HC) were shown as median by the box plot (The age and BMI were adjusted by the partial correlation test). The top 10 genera (**c**) and top 15 species (**d**) between the OA patients and HCs were showed (The age and BMI were adjusted by the partial correlation test, Additional file 1: Table S4). *p* < 0.05 was thought to be significant which was labeled as red

The species with a *p* value of less than 0.05 between the OA patients and their controls were defined as significantly different species since only *Dorea longicatena* pass the significance threshold (Q value < 0.05) after multiple testing corrections by Benjamini–Hochberg in our study. Using *p* < 0.05 as the criterion for significant difference, the gut microbiome between two groups were significantly different (*p* = 0.0418, Additional file 1: Table S1b). The gut microbial richness and diversity were decreased in OA patients both at genus (Fig. 1a, richness; Fig. 1b, α-diversity, *p* = 0.0074) and species (Additional file 1: Figure S1a) levels.

To detailedly characterize the difference, firstly we have chosen the top high abundant phyla (top 5, Additional file 1: Figure S3), genera (top 10, Fig. 1c) and species (top 15, Fig. 1d) according to the mean relative abundance in all samples. *Firmicutes* was evidently low in OA (*p* = 0.0023). Several genera including *Subdoligranulum* (*p* = 0.0413), *Ruminococcus* (*p* = 0.0012), and *Faecalibacterium* (*p* = 0.0082) were obviously decreased in OA while the *Bacteroides* was higher in OA. At species level, *B. longum*, *F. prausnitzii* (*p* = 0.0082), and *Subdoligranulum unclassified* (*p* = 0.0388) were lower in OA, while *Alistipes onderdonkii* (*A. onderdonkii*, *p* = 0.0196) was distinctly higher in OA.

**Fig. 2:**
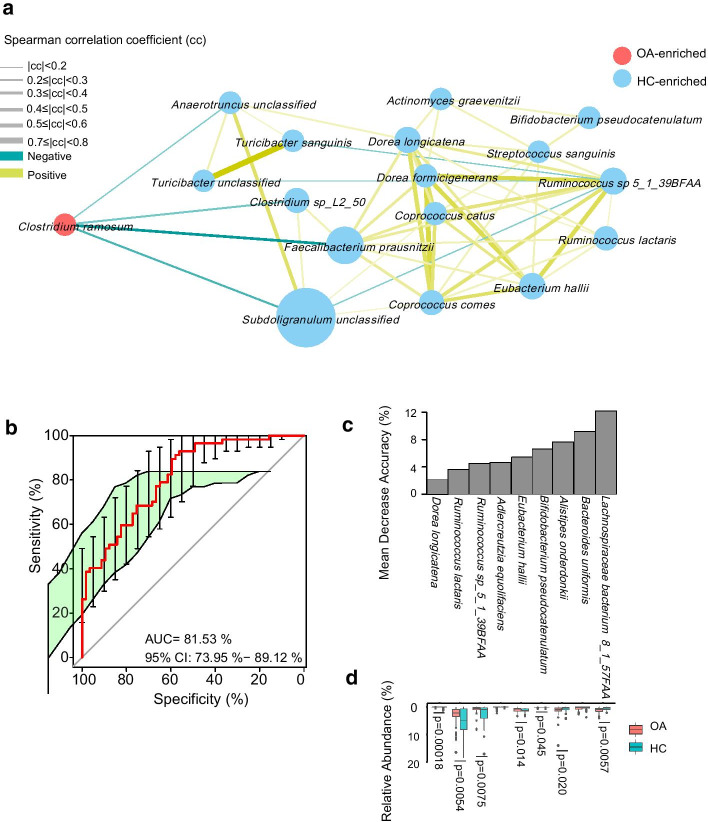
Deferentially enriched species between the OA patients and their controls. For selected different species, the orientation of enrichment was determined by partial correlation test (*p* < 0.05, Additional file 1: Table S4). Size of the nodes consistent with the relative abundance of species. Species were colored according to enrichment. Edges between nodes indicated positive (ginger) and negative (blue) Spearman’s correlation. The line thickness represents the correlation coefficient which calculated according to the samples under comparison (**a**). Receiver operating characteristic curve (ROC) according to 114 samples of the discovery set from 57 OA patients and 57 HCs (green line) calculated by Cross-validated random forest models. Area under ROC (AUC) and the 95% confidence interval are also shown (**b**). The height of the column indicates the contribution of the species to the discriminative model (**c**). The relative abundance and significance of 9 species in OA classifier (**d**)

Secondly, we evaluated the different species, genera and phyla in the two groups by Wilcoxon rank-sum test (Additional file 1: Table S4). Among all the different species, *Clostridium ramosum* (*C. ramosum*), was enriched in the OA patients while the controls were enriched in *Bifidobacterium pseudocatenulatum* (*B. pseudocatenulatum*) and *Ruminococcus lactaris* (*R. lactaris*) (Fig. 2a). To illustrate the ecosystem interaction at species level, we constructed a species network to depict the co-occurrence correlation between the OA-associated gut bacteria (Fig. 2a). Controls-enriched species were more interconnected than OA-enriched species (Spearman’s correlation coefficient <  − 0.3 or > 0.3, *p* < 0.05). Notably, more beneficial species including *B. pseudocatenulatum*, *F. prausnitzii*, *Dorea longicatena* (*D. longicatena*), *R. lactaris*, *Eubacterium hallii* (*E. hallii*) etc. were enriched in healthy controls while *C. ramosum* was enriched in the OA patients.

### The functional modules alteration

To evaluate the diversity in metabolic potential and anaerobic fermentation capacity encoded in metagenomic sequences, the GMMs were used [23]. We have compared the differences of GMMs by Wilcoxon rank-sum test and twenty-five GMMs were found to have a p value of less than 0.05, while there was no significance between the OA patients and their controls after multiple testing corrections by Benjamini–Hochberg for these GMMs. So here we used *p* < 0.05 as the criteria for significance. Among the 25 different GMMs, five GMMs including tryptophan degradation, glutamine degradation, tyrosine degradation, propionate production and acetate to actetyl-CoA were more active in the OA patients. GMMs such as triacylglycerol degradation, glycerol degradation, pentose phosphate pathway, acetyl-CoA to acetate, fructose degradation and glycolysis were higher in the healthy controls, which suggested that the gut microbiome of the OA patients were unbalanced and their healthy controls’ gut microbiome has stronger ability to metabolize nutrients (Table 1).

**Table 1:** Differentially enriched gut metabolic modules (GMMs) in OA patients and healthy controls (adjusted the age and BMI by Partial correlation test, *P* value < 0.05); whole GMMs please refer to Additional file 1: Table S5)

GMMs	*p* value	Q value	Enrichment
Tryptophan degradation	0.0021	0.0949	Healthy Controls < OA patients
Glutamine degradation I	0.0048	0.0949	Healthy Controls < OA patients
Tyrosine degradation II	0.0191	0.1462	Healthy Controls < OA patients
Propionate production III	0.0194	0.1562	Healthy Controls < OA patients
Acetate to acetyl-coa	0.0391	0.1768	Healthy Controls < OA patients
Triacylglycerol degradation	0.0033	0.0949	OA patients < Healthy Controls
Formate conversion	0.0048	0.0991	OA patients < Healthy Controls
Glycerol degradation I	0.0060	0.0982	OA patients < Healthy Controls
Bifidobacterium shunt	0.0060	0.0949	OA patients < Healthy Controls
Glycerol degradation II	0.0061	0.0949	OA patients < Healthy Controls
Pentose phosphate pathway (non-oxidative branch)	0.0066	0.0949	OA patients < Healthy Controls
Methionine degradation I	0.0081	0.1520	OA patients < Healthy Controls
Homoacetogenesis	0.0084	0.0949	OA patients < Healthy Controls
Methionine degradation II	0.0192	0.1562	OA patients < Healthy Controls
Methanogenesis from carbon dioxide	0.0233	0.1768	OA patients < Healthy Controls
Cysteine biosynthesis/homocysteine degradation	0.0241	0.1768	OA patients < Healthy Controls
Acetyl-coa to acetate	0.0252	0.1591	OA patients < Healthy Controls
Fructose degradation	0.0343	0.1520	OA patients < Healthy Controls
Serine degradation	0.0365	0.1768	OA patients < Healthy Controls
Methanogenesis-methyl-com	0.0387	0.2107	OA patients < Healthy Controls
Glycolysis (preparatory phase)	0.0445	0.1878	OA patients < Healthy Controls
Fucose degradation	0.0454	0.2211	OA patients < Healthy Controls
Glutamine degradation II	0.0467	0.2107	OA patients < Healthy Controls
Glycolysis (pay-off phase)	0.0476	0.1768	OA patients < Healthy Controls
Hydrogen metabolism	0.0496	0.3106	OA patients < Healthy Controls

The *p* value was calculated by Wilcoxon rank sum test, the Q value was the *p* value adjusted by Benjamini–Hochberg. Enrichment means the relative abundance of the related GMMs in the healthy controls and the OA patients

### Gut microbial species characteristic of osteoarthritis

To identify gut bacterial species associated with OA and evaluate their diagnostic values, we constructed a set of random forest disease classifiers based on gut species. We performed a five-fold cross-validation procedure ten times on 57 OA patients and 57 controls. Nine gut species reached the lowest classifier error in the random forest cross validation, and the area under the receiver operating characteristic curve (AUC) of the model was 0.81 (Fig. 2b). The importance and enrichment of these species-based markers are shown in Fig. 2c. This microbial based classifier was corrected by age and BMI. A significant decrease in *D. longicatena*, *R. lactaris*, *Ruminococcus sp_5_1_39BFAA*, *E. hallii*, *B. pseudocatenulatum* and an obvious increase in that of *A. onderdonki* and *Lachnospiraceae bacterium8_1_57FAA* were observed in the OA patients, which were supposed to be biomarkers of OA.

## Discussion

In this study, high quality reads from a previous published paper by Xie et al. [20] were matched to the newest human gut microbial gene set [20]. Significant changes in identified genes, phylogenies and functional modules in the gut microbiome were demonstrated for OA patients and their healthy controls. It's worth noting that we used *p* < 0.05 as the significance criteria for different species and GMMs between the OA patients and their healthy controls since there was no significant difference between the two groups after multiple testing corrections by Benjamini–Hochberg except for *Dorea longicatena* (q = 0.028). This means that gut microbial differences do exist between groups but are not large, which might be due to the limited sample size to detect statistically significant differences in the gut microbiota composition in this study.

Gut microbial richness and diversity was obviously low in OA, along with a decrease in some bacteria including *A. onderdonkii*, *Subdoligranulum* spp., *R. lactaris*, *B. longum*, *B. pseudocatenulatum* and *F. prausnitzii*, as well as an increase in some pathogenic microbes, such as *C. ramosum*. *A. onderdonkii* might be an indicator of gut disorder [27]. *B. longum* is able to regulate the immune system and has been used to treat ulcerative colitis as a constitute in VSL#3 [28]. *R. lactaris* is a producer of metabolites including acetate, lactate, formate, succinate, cobalamin, pyridoxine, and thiamine (https://microbiomeprescription.com/library/details?taxon=46228). *F. prausnitzii* is an anti-inflammatory species by producing butyrate for a healthy gut [29]. *Subdoligranulum* is a relative of *F. prausnitzii* and is beneficial for human health [30]. *B. pseudocatenulatum* can ameliorate neuroendocrine alterations associated with an exaggerated stress response and anhedonia in bbese mice [26]. *C. ramosum* is infrequently a cause of pathologic infection [31]. In addition, the Firmicutes/Bacteroidetes (F/B) ratio is widely accepted to have an important influence in maintaining normal gut homeostasis and an decrease in the F/B ratio is usually reported in inflammatory diseases such as IBD. In our study, we have also observed an increase in Bacteriodetes and decrease in Firmicutes, which means a decline in the F/B ratio, is consistent with the other studies. These results suggested that a decrease in the diversity and relative abundance of some beneficial bacteria, as well as an increase of the relative abundance of pathogenic species might be risk factors for OA.

In addition to the compositional changes, the functional changes to access the metabolic potential and anaerobic fermentation capacity of the gut microbiome were also observed between the OA patients and their controls in our study. Significantly higher degradation in tryptophan, glutamine and tyrosine, as well as propionate production were observed in the OA patients. Tryptophan can be mainly metabolized to serotonin [32] and pyruvate, a short-chain fatty acid (SCFA) producer [33] and a higher degradation in it means higher levels of serotonin and SCFAs in OA patients, which means the gut microbiota can self-regulate under some inflammatory conditions. Glutamine is a major substrate for intestinal cells to protect cells against apoptosis and cellular stresses [34] and an increase in the glutamine degradation meant a low level of glutamine in the OA patients which might accentuate OA. Tyrosine can be degraded by some gram-positive bacteria to pyruvate and succinate to further produce SCFA [7]. In addition, tyrosine can inhibit human gut decarboxylase to produce dopamine [35]. Higher levels of acetate and low levels of formate and propionate in healthy controls suggested that different kinds of SCFA might have different influence on OA. The methanogenesis of methanogens in the gut can reduce intestinal gas accumulation, and compete with other symbiosis microorganisms to stabilize the gut balance and keep away from irritable bowel syndrome [36] and colorectal cancer [37]. These observations suggested that once the human body was under some inflammatory conditions, the gut microbial composition will change and their functions will antagonize to keep the body at a relatively healthier level.

Finally, we constructed a microbial classifier containing 9 different species by random forest model with an AUC of 0.81 to identify the OA patients. Regrettably, our prediction model was only reliable in the older female adults and was not verified in other cohorts. However, our results at least demonstrated the fact that there are some key species varied between the older female patients with OA and their healthy controls, suggesting that the isolation and in vitro/in vivo functional verification of these signature microbial strains might benefit the older females with OA.

## Conclusions

Our results revealed the changes in gut microbial composition and function between the older female patients with OA and their healthy controls. Some species and GMMs with a p value less than 0.05 were identified between the two groups, but most of them had no significant difference after multiple testing correction by Benjamini–Hochberg which might be caused by limited sample size. Based on the different species with *p* < 0.05, we have constructed a OA classifier including 9 species with an AUC of 0.81. The dysbiosis of the gut microbiome in the OA patients reminded us that manipulation of the gut microbiota by probiotics or prebiotics might be an effective strategy to regulate and maintain the homeostasis of the gut microbiota to mitigate symptoms of OA and improve the life quality of the older females.

## Supplementary Information

**Additional file 1.** Supplementary material contains tables, figures, and code used in the study.

## Data Availability

The datasets used to analyse for this study can be found in the European Bioinformatics Institute (EBI) with the accession ID ERP010708 or in the China National Genebank (CNGB) with a program ID CNPhis0000107.

## Abbreviations

OA: Osteoarthritis
LPS: Lipopolysaccharide
WOMAC: The Western Ontario and McMaster Universities Arthritis Index
HFD: High fat diet
OARSI: Osteoarthritis cartilage histopathology assessment system
AUC: Area under curve
UK: United Kingdom
BMI: Body mass index
KEGG: Kyoto encyclopedia of genes and genomes
KO: KEGG Orthology
MZ: Monozygotic
DZ: Dizygotic
GMM: Gut metabolic module
PERMANOVA: Permutational Multivariate Analysis of Variance
FDR: False Discovery Rates
SCFA: Short-chain fatty acid
*F. prausnitzii*: *Faecalibacterium prausnitzii*
*B. longum*: *Bifidobacterium longum*
*A. onderdonkii*: *Alistipes onderdonkii*
*C. ramosum*: *Clostridium ramosum*
*B. pseudocatenulatum*: *Bifidobacterium pseudocatenulatum*
*R. lactaris*: *Ruminococcus lactaris*
*D. longicatena*: *Dorea longicatena*
*E. hallii*: *Eubacterium hallii*
