# Supplementary material for: Dysbiosis of the gut microbiome is a risk factor for osteoarthritis in older female adults: a case control study
Source: BMC Bioinformatics. 2021 Jun 3;22:299. doi: 10.1186/s12859-021-04199-0 (PMC8173911; doi:10.1186/s12859-021-04199-0)
Supplement: Supplementary file 1 — Additional file 1. Supplementary material contains tables, figures, and code used in the study. [file 12859_2021_4199_MOESM1_ESM.zip › Additional file 8. Codes used in the study..pdf]

1.alpha_shannon.pl: To calculate the alpha diversity (Shannon index) of sample.

2.permanova.R: To evaluate the effect of clinical indexes on the gut microbiome.

3.ppcor.R: To adjust for BMI and age.

4.randomForest.R;rfcv1.R;ROC.R: The code for the random forest model, five–fold cross–validation was performed ten times using the species and functional modules abundance profiles of the two groups. The test error curves from ten trials of five–fold cross–validation were averaged.

5.get_functional_profile.pl: To calculate the KO abundance from gene abundance.

1.alpha_shannon.pl: To calculate the alpha diversity (Shannon index) of sample.

```
use warnings;
use strict;

die "perl $0 [*.in] [*.out]\n" unless @ARGV == 2;

my ($in_f, $out_f) = @ARGV;
die "Overlap In-Output...\n" if $in_f eq $out_f;
### print STDERR "Program $0 Start...\n";

my (@gene, @sum, @shannon) = ();

open IN, $in_f or die $!;
chomp(my $h=<IN>);
my @head = split /\s+/, $h;
shift @head;
while(<IN>){
	chomp;
	my @s = split /\s+/;
	shift @s;
	for(0..$#s){
		next if $s[$_]==0;
		### $s[$_]/=1e6;
		$gene[$_]++;
		$sum[$_] += $s[$_];
		$shannon[$_] -= ($s[$_]) * log($s[$_]);
	}
}
close IN;

open OT, ">$out_f" or die $!;
for(0..$#head){
	print STDERR "SUM: $head[$_]\t$sum[$_]\n";
	print OT "$head[$_]\t$gene[$_]\t$shannon[$_]\n";
}
close OT;

print STDERR "Program End...\n";
############################################################
sub function {
	return;
}
############################################################
```

2.permanova.R: To evaluate the effect of clinical indexes on the gut microbiome.

```
args <- commandArgs(T)
if (length(args) != 5) {
    stop("Rscript permanova.R [input1] [input2] [input3] [input4] [prefix]\ninput1: Distance Matrix\ninput2: Phenotype\ninput3: colClasses of Phenotype\ninput4: permutation times\nprefix: eg: Bray Euclid\nmake sure sample of phe.pr and dist.pr have the same rownames")
}

dist.dir <- args[1]
phe.dir <- args[2]
cl.dir <- args[3]
ps <- as.numeric(args[4])
prefix <- args[5]
out.dir <- paste(prefix, "_adonis.txt", sep = "")

library(vegan)

dist.pr <- read.table(dist.dir)
cl.pr <- read.table(cl.dir, stringsAsFactors = F)
phe.pr <- read.table(phe.dir, colClasses = c(NA, cl.pr[, 1]))
phe.cn <- colnames(phe.pr)

dist.rn <- rownames(dist.pr)
phe.rn <- rownames(phe.pr)

rn <- intersect(phe.rn, dist.rn)

flag <- pmatch(rn, dist.rn)
dist.pr <- dist.pr[flag, flag, drop = F]
flag <- pmatch(rn, phe.rn)
phe.pr <- phe.pr[flag, , drop = F]

out.cn <- list("phenotype", "SampleNum", "Df", "SumsOfSqs", "MeanSqs", "F.Model", "R2", "Pr(>F)")
write.table(out.cn, out.dir, quote = F, sep = "\t", col.names = F, row.names = F)

for (i in 1:ncol(phe.pr)) {
    phe <- phe.pr[, i]
    flag <- which(!is.na(phe))
    len <- length(flag)
    phe <- phe[flag]

    if (len == 0 | length(unique(phe)) == 1) {
        out <- list(phe.cn[i], len, rep(NA, 6))
        write.table(out, out.dir, quote = F, sep = "\t", col.names = F, row.names = F, append = T)
```

```
        next
    }

    set.seed(0)
    dist <- as.dist(dist.pr[flag, flag, drop = F])
    res <- adonis(dist ~ phe, permutations = ps)

    out <- list(phe.cn[i], len, res$aov.tab[1, ])
    write.table(out, out.dir, quote = F, sep = "\t", col.names = F, row.names = F, append = T)
}
```

3.ppcor.R: To adjust for BMI and age.

```
library(ppcor)
infile_phe<-read.table('updated.phe.txt',sep='\t',row.names = 1,header = T)
infile_dat<-t(read.table('p_merged_abundance_table.txt',sep='\t',row.names = 1,header = T))
out_dat<-matrix(NA,ncol(infile_dat),3)
for (i in (1:ncol(infile_dat))) {
    y.data<-data.frame(infile_phe$sample_time,infile_dat[,i],infile_phe$sex, infile_phe$age)
    a<-pcor.test(y.data$infile_phe.sample_time,y.data[,2],y.data[,c(3:4)],method = "spearman")
    out_dat[i,]<-c(a$estimate,a$p.value,a$statistic)
}
rownames(out_dat) <- colnames(infile_dat)
colnames(out_dat)<-c('estimate','pvalue','statistic')
write.table(out_dat,'phylum.spcor.sex.age.txt',sep='\t')

y.data<-data.frame(infile_phe$sample_time,infile_phe$ge.shannon,infile_phe$sex, infile_phe$age)
a<-pcor.test(y.data$infile_phe.sample_time,y.data[,2],y.data[,c(3)],method = "spearman")
c(a$estimate,a$p.value,a$statistic)
```

4.randomForest.R;rfcv1.R;ROC.R: The code for the random forest model, five-fold cross-validation was performed ten times using the species and functional modules abundance profiles of the two groups. The test error curves from ten trials of five-fold cross-validation were averaged.

```
args <- commandArgs(T)

cat("make sure:\n        y and x must have the same sample id\n        train_y must have only two levels and will change to 0 and 1\n        test_y which not in train_y will change to 2\n        set marker_num 0 to compute automaticity\nnote:if x and y have different levels could lead to errors\n")

if (length(args) != 10) {
    stop("Rscript *.R [train_x] [train_y] [test_x] [test_y] [cv_fold] [cv_step] [cv_time] [marker_num] [prefix] [seed]\n")
}
```

```r
train.x <- args[1]
train.y <- args[2]
test.x <- args[3]
test.y <- args[4]
cv.fold <- as.numeric(args[5])
cv.step <- as.numeric(args[6])
cv.time <- as.numeric(args[7])
marker.num <- as.numeric(args[8])
prefix <- args[9]
seeds<-as.numeric(args[10])
# package
library(randomForest)

args <- commandArgs(F)
SD <- dirname(sub("--file=", "", args[grep("--file=", args)]))
# function
source('rfcv1.R')
source('ROC.R')

# data
train.x <- t(read.table(train.x))
train.y <- as.factor(read.table(train.y)[, 1])
train.l <- levels(train.y)
levels(train.y) <- 0:1
print(train.y)
print(train.l)

test.x <- t(read.table(test.x))
test.y <- as.factor(read.table(test.y)[, 1])
test.l <- levels(test.y)
levels(test.y) <- pmatch(test.l, train.l) - 1
test.y <- factor(test.y, 0:2)

# crossvalidation
pdf.dir <- paste0(prefix, "_randomForest.pdf")
pdf(pdf.dir, width = 35, height = 7)
par(mfrow = c(1, 6))

set.seed(seeds)
train.cv <- replicate(cv.time, rfcv1(train.x, train.y, cv.fold = cv.fold, step = cv.step), simplify = F)
error.cv <- sapply(train.cv, "[[", "error.cv")
error.cv.rm <- rowMeans(error.cv)
# id <- error.cv.rm < min(error.cv.rm) + diff(range(error.cv.rm))/20
```

```
id <- error.cv.rm < min(error.cv.rm) + sd(error.cv.rm)
error.cv[id, ]
if (marker.num == 0) {
    marker.num <- min(as.numeric(names(error.cv.rm)[id]))
}
matplot(train.cv[[1]]$n.var, error.cv, type = "l", log = "x", col = rep(1, cv.time), main = paste("select",
marker.num, "Vars"), xlab = "Number of vars",
    ylab = "CV Error", lty = 1)
lines(train.cv[[1]]$n.var, error.cv.rm, lwd = 2)
abline(v = marker.num, col = "pink", lwd = 2)

# pick marker by corossvalidation
marker.t <- table(unlist(lapply(train.cv, function(x) {
    lapply(x$res, "[", 1:marker.num)
})))
marker.t <- sort(marker.t, d = T)
names(marker.t) <- colnames(train.x)[as.numeric(names(marker.t))]
marker.dir <- paste0(prefix, "_marker.txt")
write.table(marker.t, marker.dir, col.names = F, sep = "\t", quote = F)
marker.p <- names(marker.t)[1:marker.num]

# train model
set.seed(0)
train.rf <- randomForest(train.x[, marker.p], train.y, importance = T)
imp<-importance(train.rf)
write.table(importance(train.rf),paste0(prefix, "_MeanDecreaseAccuracy.txt"),sep = '\t')
#imp<-read.table('./result/test1_MeanDecreaseAccuracy.txt',header=T,row.names = 1,sep='\t')
imp<-imp[order(imp[,5]),]
par(mar=c(8,4,4,4))
barplot(imp$MeanDecreaseAccuracy,ylab ='MeanDecreaseAccuracy',xlab = '',space =0)
text(1:length(imp$MeanDecreaseAccuracy)-1, -0.1, labels =imp$ano,pos=4,srt = 270, xpd = T,cex = 0.6)
train.p <- predict(train.rf, type = "prob")
boxplot(train.p[, 2] ~ train.y, col = 2:3, main = "Probability", names = train.l)
pr.dir <- paste0(prefix, "_train_probability.txt")
write.table(train.p[, 2], pr.dir, sep = "\t", quote = F, col.names = F)

# train ROC
plot_roc(train.y, train.p[, 2])

# test predict
test.p <- predict(train.rf, test.x, type = "prob")
pr.dir <- paste0(prefix, "_test_probability.txt")
write.table(test.p[, 2], pr.dir, sep = "\t", quote = F, col.names = F)
```

```
# predict plot
p.col <- ifelse(is.na(test.y), 4, as.numeric(test.y) + 1)
plot(rank(test.p[, 2]), test.p[, 2], col = p.col, pch = 16, xlab = "", ylab = "Probability", main = "Testset")
txt <- train.l
if (length(test.l) > 2) {
    txt <- c(txt, "the rest")
}
legend("bottomright", txt, col = 2:4, pch = 16)
abline(h = 0.5)

# test ROC
plot_roc(test.y, test.p[, 2])
dev.off()
```

6.get_KO_profile.pl: To calculate the KO abundance from gene abundance.

```
#! /usr/bin/perl

=head1 Program: get_function_profile.pl

=head1 Description: This program use to get function profile from gene profile and function annotation

=head1

        Usage: perl get_function_profile.pl [options]

        Options:
        -k    <str>      catalog[KO name; gene number; gene]
        -f    <str>      gene profile file
        -o    <str>      output dir
        gene is the same as the fist col of gene profile

=head1

=cut

use strict;
use warnings;

use Getopt::Long;

#initialize some parameters
our ($corr_k, $profile_gene, $outdir);    # input
our (%profile_d);                         # output
our (%anno, %profile, %ss, @sample);      # anno sample profile
```

```
our (@s, $i);                                    # else

GetOptions(
        "k=s" => \$corr_k,
        "f=s" => \$profile_gene,
        "o=s" => \$outdir,
);
#get the introduction information
die `pod2text $0` if (!$corr_k || !$profile_gene);

$outdir ||= ".";
$outdir =~ s/\/$//;

my $pwd = $ENV{'PWD'};

$outdir = "$pwd/$outdir" if ($outdir !~ /^\//);

$corr_k = "$pwd/$corr_k" if ($corr_k && $corr_k !~ /^\//);

$profile_gene = "$pwd/$profile_gene" if ($profile_gene !~ /^\//);

`mkdir -p $outdir` unless (-e $outdir);

#profile result file
$profile_d{k1} = $outdir.'/accumulation.pr';
$profile_d{k2} = $outdir.'/sample.abundance.total.rate';
$profile_d{k3} = $outdir.'/normalization.pr';

#anno
if ($corr_k =~ /\.gz$/) {
        open FA, "gzip -dc $corr_k |" or die $!;
} else {
        open FA, $corr_k or die $!;
}

<FA>;
while (<FA>) {
        chomp;
        @s = split /\t/;
        $anno{k}{$s[0]} = 1;
        for ($i = 2; $i<= $#s; ++$i) {
                $anno{$s[$i]}{$s[0]} = 1;
        }
}
```

```
close FA;

#gene profile
open GP, $profile_gene or die "can't read $profile_gene:$!\n";
$_ = <GP>;
chomp;
@sample = split(/\t/, $_);

while (<GP>) {
	chomp;
	@s = split /\t/;
	if (exists $anno{$s[0]}) {
		for my $anno (keys %{$anno{$s[0]}}) {
			for ($i = 1; $i <= $#s; ++$i) {
				$profile{k}{$anno}{$i} += $s[$i];
			}
		}
	}
}
close GP;

#profile
open PP, ">$profile_d{k1}" or die "can't write $profile_d{k1}:$!\n";
print PP join("\t", @sample), "\n";

foreach my $anno (sort keys %{$profile{k}}) {
	my $anno_nospace = $anno;
	$anno_nospace =~ s/\s+/\_/g;
	print PP $anno_nospace;
	for ($i = 1; $i <= $#sample; ++$i) {
		print PP "\t$profile{k}{$anno}{$i}";
	}
	print PP "\n";
}
close PP;

open PP, ">$profile_d{k2}" or die "can't write $profile_d{k2}:$!\n";
foreach my $anno (sort keys %{$profile{k}}) {
	for ($i = 1; $i <= $#sample; ++$i) {
		$ss{k}{$i} += $profile{k}{$anno}{$i};
	}
}
for ($i = 1; $i <= $#sample; ++$i) {
	print PP "$sample[$i]\t$ss{k}{$i}\n";
```

```
}
close PP;


open PP, ">$profile_d{k3}" or die "can't write $profile_d{k3}:$!\n";
print PP join("\t", @sample), "\n";

foreach my $anno (sort keys %{$profile{k}}) {
	my $anno_nospace = $anno;
	$anno_nospace =~ s/\s+/\_/g;
	print PP $anno_nospace;
	for ($i = 1; $i <= $#sample; ++$i) {
		print PP "\t", $profile{k}{$anno}{$i}/$ss{k}{$i};
	}
	print PP "\n";
}
close PP;
```
