# Supplementary material for: Dysbiosis of the gut microbiome is a risk factor for osteoarthritis in older female adults: a case control study
Source: BMC Bioinformatics. 2021 Jun 3;22:299. doi: 10.1186/s12859-021-04199-0 (PMC8173911; doi:10.1186/s12859-021-04199-0)
Supplement: Supplementary file 1 — Additional file 1. Supplementary material contains tables, figures, and code used in the study. [file 12859_2021_4199_MOESM1_ESM.zip › Illustrations for the additional file.docx]

**Illustrations of the additional files**

1. Additional file 1. Supplemental Table S1, The clinical phenotypes recorded for all the participants (Table S1a) and the PERMANOVA results of the clinical phenotypes to the gut microbiome at gene level (Table S1b).
2. Additional file 2. Supplemental Table S2. The high quality data from metagenomic shotgun sequencing of all the samples.
3. Additional file 3. Supplemental Table S3. The count and diversity data (from Shannon index) for all the samples at genus, species and KO level.
4. Additional file 4. Supplemental Table S4. The differences between the osteoarthritis patients and healthy controls at phylum (Table S4a), genus (Table S4b) and species (Table S4b). The profile at each level were calculated by Metaphlan2, the differences between the two groups at each level were calculated by Wilcoxon rank sum test.
5. Additional file 5. Supplemental Table S5. The differences of the gut microbial function (gut metabolic modules, GMMs) between the two groups which calculated by Wilcoxon rank sum test.
6. Additional file 6. Supplemental Figure S1. Richness and alpha diversity in species level. The species count (a) and diversity (b) in the healthy group (HC) were higher than those in the osteoarthritis patients (OA). The results were adjusted by the age and body mass index (BMI) by Partial correlation test.
7. Additional file 7. Supplemental Figure S2. The top 5 phyla showed a difference between the osteoarthritis patients (OA) and the healthy controls (HC). The results were adjusted by the age and body mass index (BMI) by Partial correlation test, and the data used to make this figure were from supplemental Table S4.
8. Additional file 8. The codes used in the study.
