# Supplementary figures and images for: Dysbiosis of the gut microbiome is a risk factor for osteoarthritis in older female adults: a case control study
Source: BMC Bioinformatics. 2021 Jun 3;22:299. doi: 10.1186/s12859-021-04199-0 (PMC8173911; doi:10.1186/s12859-021-04199-0)

a

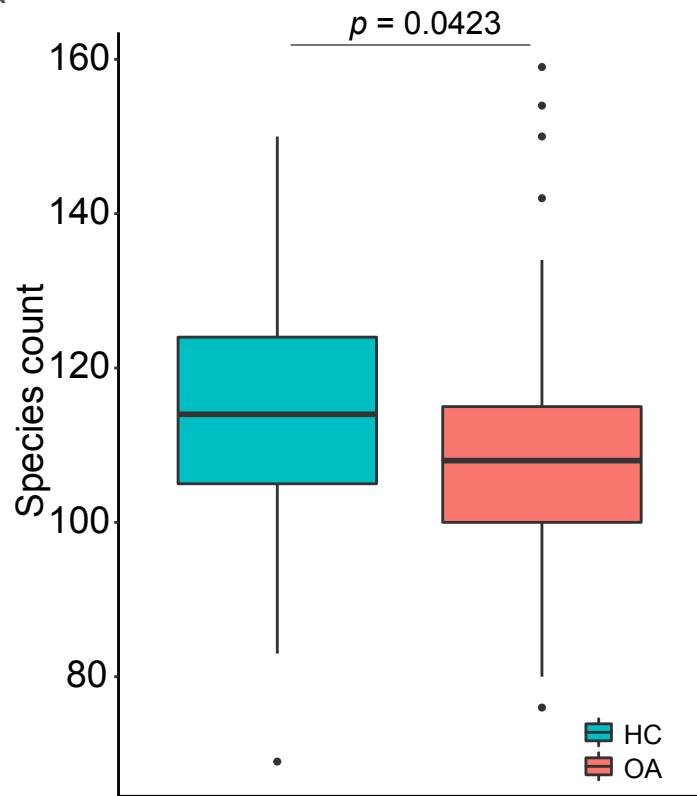

b

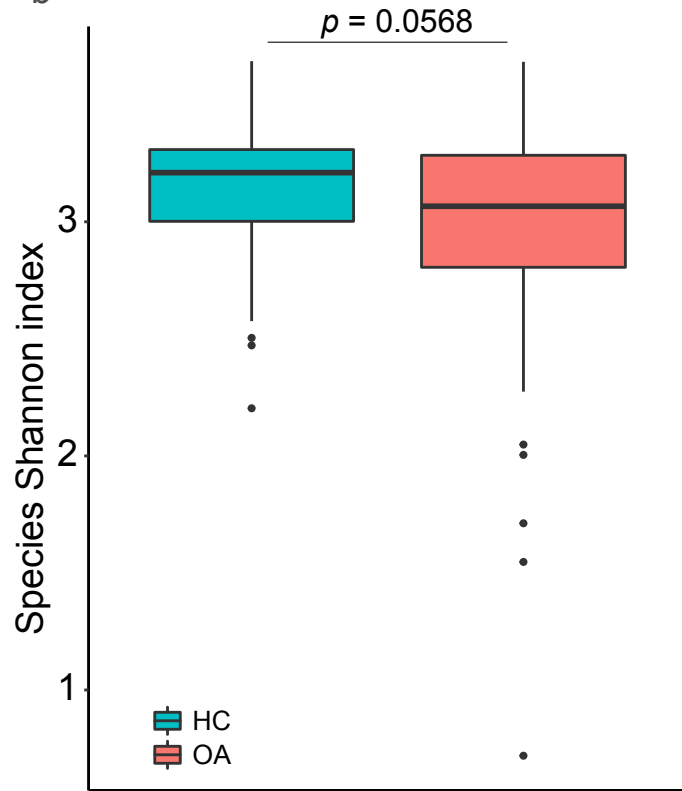

Supplement: Supplementary file 1 — Additional file 1. Supplementary material contains tables, figures, and code used in the study. [file 12859_2021_4199_MOESM1_ESM.zip › Additional file 6. Figure S1.pdf]

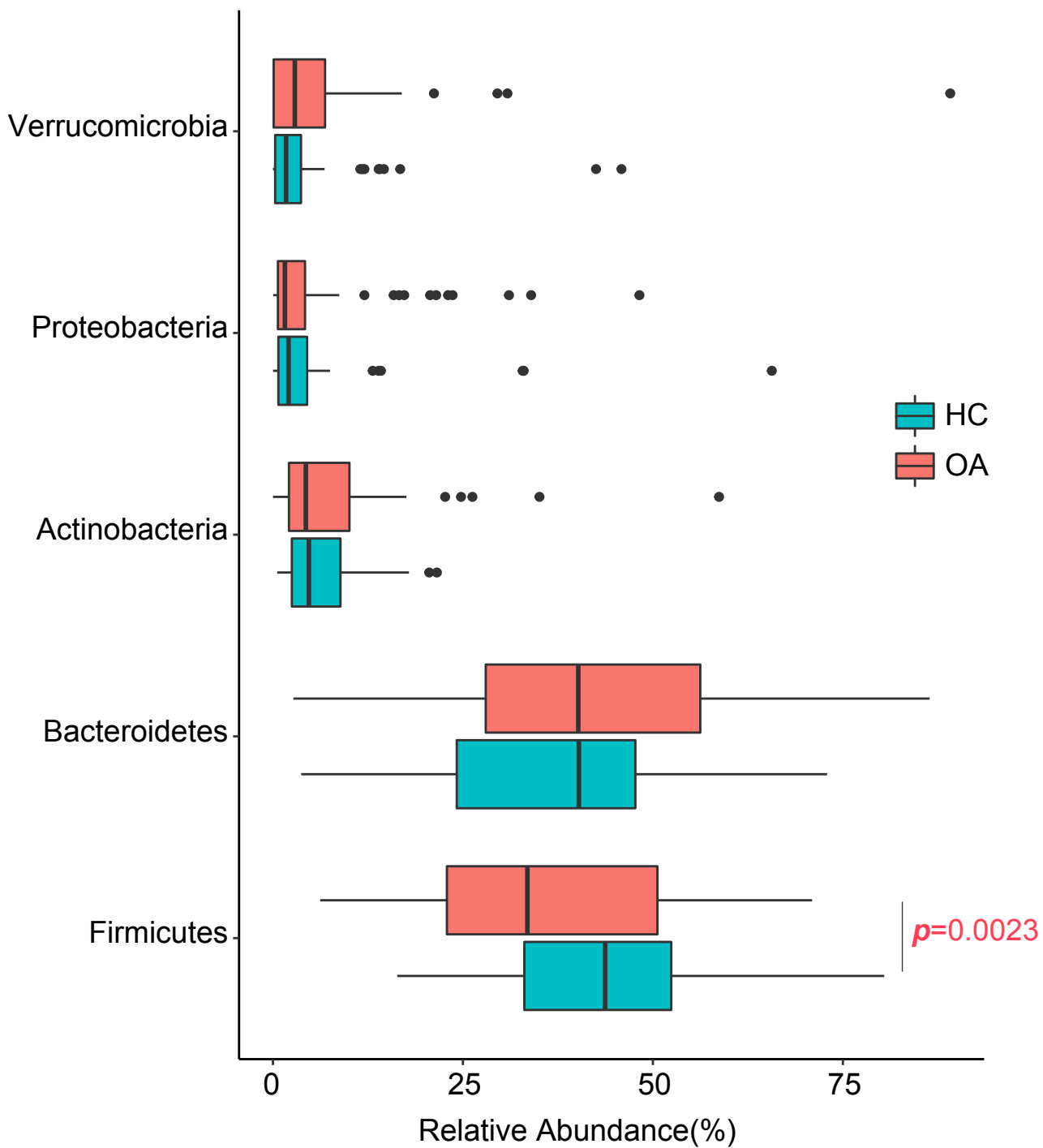

Supplement: Supplementary file 1 — Additional file 1. Supplementary material contains tables, figures, and code used in the study. [file 12859_2021_4199_MOESM1_ESM.zip › Additional file 7. Figure S2.pdf]
